# *NOS2* Is Critical to the Development of Emphysema in Sftpd Deficient Mice but Does Not Affect Surfactant Homeostasis

**DOI:** 10.1371/journal.pone.0085722

**Published:** 2014-01-21

**Authors:** Lars Knudsen, Elena N. Atochina-Vasserman, Chang-Jiang Guo, Pamela A. Scott, Beat Haenni, Michael F. Beers, Matthias Ochs, Andrew J. Gow

**Affiliations:** 1 Institute of Anatomy, University of Bern, Bern, Switzerland; 2 Institute of Functional and Applied Anatomy, Hannover Medical School, Hannover, Germany; 3 Biomedical Research in Endstage and Obstructive Lung Disease Hannover (BREATH), Member of the German Center for Lung Research (DZL), Hannover, Germany; 4 Pulmonary, Allergy and Critical Care Division, University of Pennsylvania, Philadelphia, Pennsylvania, United States of America; 5 Department of Pharmacology & Toxicology, Rutgers University, Piscataway, New Jersey, United States of America; 6 REBIRTH Cluster of Excellence, Hannover, Germany; Chinese Academy of Sciences, China

## Abstract

**Rationale:**

Surfactant protein D (SP-D) has important immuno-modulatory properties. The absence of SP-D results in an inducible NO synthase (iNOS, coded by NOS2 gene) related chronic inflammation, development of emphysema-like pathophysiology and alterations of surfactant homeostasis.

**Objective:**

In order to test the hypothesis that SP-D deficiency related abnormalities in pulmonary structure and function are a consequence of iNOS induced inflammation, we generated SP-D and iNOS double knockout mice (DiNOS).

**Methods:**

Structural data obtained by design-based stereology to quantify the emphysema-like phenotype and disturbances of the intracellular surfactant were correlated to invasive pulmonary function tests and inflammatory markers including activation markers of alveolar macrophages and compared to SP-D (Sftpd^−/−^) and iNOS single knockout mice (NOS2^−/−^) as well as wild type (WT) littermates.

**Measurements and Results:**

DiNOS mice had reduced inflammatory cells in BAL and BAL-derived alveolar macrophages showed an increased expression of markers of an alternative activation as well as reduced inflammation. As evidenced by increased alveolar numbers and surface area, emphysematous changes were attenuated in DiNOS while disturbances of the surfactant system remained virtually unchanged. Sftpd^−/−^ demonstrated alterations of intrinsic mechanical properties of lung parenchyma as shown by reduced stiffness and resistance at its static limits, which could be corrected by additional ablation of NOS2 gene in DiNOS.

**Conclusion:**

iNOS related inflammation in the absence of SP-D is involved in the emphysematous remodeling leading to a loss of alveoli and associated alterations of elastic properties of lung parenchyma while disturbances of surfactant homeostasis are mediated by different mechanisms.

## Introduction

Surfactant Protein D (SP-D) is a member of the superfamily of collectins, and participates in innate host defense with well-established immunomodulatory properties [1], [2]. Potential mechanisms and receptors enabling this protein to either attenuate or enhance inflammatory processes, depending upon the local milieu, have been revealed [3], [4], [5]. Mice deficient in SP-D (Sftpd^−/−^) develop an early onset emphysematous phenotype, hypertrophy and hyperplasia of alveolar type II cells (AE2 cells) and disturbances of surfactant homoeostasis such as an alveolar lipoproteinosis and an increased number of lamellar bodies per type II airway epithelial cells (AE2 cell) [6], [7], [8], [9], [10]. Accumulation of foamy appearing alveolar macrophages and peribronchial and perivascular infiltrates are typical findings, which precede lung remodeling [6], [11]. The precise mechanisms of this pathology are not clear, although replacement therapy with structural mutants of SP-D or inhibition of the inducible isoform of Nitric Oxide Synthase (iNOS, coded by NOS2 gene) alleviate certain aspects [12], [13], [14]. These studies have established that a chronic inflammatory state, involving iNOS, is associated with SP-D deficiency.

Aberrant alveolar macrophage activity is a component of the inflammation that occurs within Sftpd^−/−^ mice. Along with increased iNOS, there is enhanced macrophage production of reactive oxygen species (ROS) and chemokines; suggesting that SP-D deficiency results in an increased local flux of oxidative-nitrosative stress in the distal lung [11], [15], [16], [17]. Increased iNOS activity occurs in both macrophages and AE2 cells within chronic obstructive pulmonary disease (COPD) [18], [19]. Oxidative-nitrosative stress regulates the activity of transcription factors involved in inflammation, such as NF-κB [20] whose function leads to increased activity of the metalloproteinases 2, 9, and 12 in Sftpd^−/−^ mice. Therefore, oxidative stress may be a key mediator of the alveolar destruction and subsequent development of an emphysematous phenotype in Sftpd^−/−^ mice [12], [15], [21].

Previously, we have examined the effects of iNOS inhibition with the inhibitor, 1400W [12]. The emphysematous phenotype that develops in Sftpd^−/−^ mice is progressive and age dependent and is associated with increased iNOS expression. Long-term inhibition of iNOS, from 3 weeks of age, reduces the progressive inflammation observed in Sftpd^−/−^ mice. 1400W treatment reduces established inflammation but not lipoproteinosis when given to 8 week old Sftpd^−/−^ mice. Furthermore, although we were able to observe alteration in chemokine expression, it was not determined whether iNOS inhibition had altered the structural and functional changes associated with loss of SP-D.

As the pathology associated within Sftpd^−/−^ mice is progressive, it is unclear at what age it is initiated. We hypothesized that the early loss of NOS2, attenuated inflammatory processes as well as structural and functional changes seen as a result of Sftpd ablation. We chose a genetic approach to further address the role of NOS2 in Sftpd related lung remodeling by developing a double knockout murine model deficient in both SP-D and iNOS (i.e. Sftpd^−/−^/NOS2^−/−^ mice). Using both morphometric and physiological endpoints, data generated with this model indicate that iNOS related inflammation in the absence of SP-D is responsible for emphysematous remodeling leading to a loss of alveoli and associated alterations of elastic properties of lung parenchyma

## Materials and Methods

### Transgenic Mouse Models

The generation of the Sftpd^−/−^ mice was previously described [6], [8], NOS2 deficient mice (NOS2^−/−^) on C57BL/6 background were purchased from Jackson Laboratories, Inc. (Bar Harbor, ME). Sftpd^−/−^ and NOS2^−/−^ mice were bred to obtain mice heterozygous for Sftpd and NOS2. Double heterozygous mice were intercrossed to generate wild type (WT), null for SP-D alone (Sftpd^−/−^) or NOS2 alone (NOS2^−/−^), or both genes Sftpd^−/−^/NOS2^−/−^ (DiNOS). WT, Sftpd^−/−^ and DiNOS mice on the C57BL/6 background were maintained and bred at the animal care facility at the Rutgers University (Piscataway, NJ). Experiments were conducted at 12 weeks of age. All protocols were reviewed and approved by the Institutional Animal Care and Use Committees of the University of Pennsylvania and Rutgers University and adhered to the principles of the National Institutes of Health Guide for the Care and Use of Laboratory Animals.

### Bronchoalveolar Lavage Fluid (BALF) Analyses

The total number of cells within BALF as well cell differential was determined. In addition large (LA) and small (SA) aggregates of surfactant, total phospholipid content within LA and SA as well as NO metabolites were determined. RT-PCR was used do quantify gene-expression markers.

### Fixation, Sampling and Processing

At the age of 12 weeks 5–6 animals were fixed by airway instillation with a constant hydrostatic pressure of 25 cm H_2_O, applying a 1.5% glutaraldehyde/1.5% paraformaldeyde mixture in 0.15M HEPES buffer and further processed for design-based stereology [22].

### Stereological Analysis

Stereological assessment is based on the ATS/ERS statement on quantitative evaluation of lung structure [23] (online supplement). To quantify phenotypic changes, surface area of alveolar epithelium and the number and number-weighted mean size of the alveoli were determined [24], [25]. The volume-weighted mean volume of alveoli, a parameter reflecting also the heterogeneity of airspace enlargement, was quantified [26]. Intracellular surfactant, as defined as the total lamellar bodies within Type II airway epithelial cells (AE2), was assessed by determining number and size of the cells and the volume fraction of lamellar bodies per cell. Absolute lamellar body volume per AE2 cell and lung was calculated [27].

### Assessment of Pulmonary Mechanics

Pulmonary mechanics were assessed on anesthetized mice as previously described [28] and detailed in the online supplement. Briefly mechanics were measured using a forced oscillation technique and resistance (*R_L_*) and elastance (*E_L_*) spectra via an empirical model.

### Statistics

*E_L_* and *R_L_* spectra were compared between genotypes using the Pearson's χ^2^ test. For significantly different spectra, parameters were compared using student's t-test. Multiple comparisons were made by one-way ANOVA and Bonferroni's Multiple Comparison Test. Stereological data were compared by Kruskal-Wallis-test and Dunn's correction for multiple comparisons. Assessment was performed by means of GraphPad Prism version 4.00 (GraphPad Software, San Diego, CA). The level of significance was p<0.05.

## Results

### NOS2 Ablation reduces BAL nitrates in DiNOS Mice

**Figure 1:**
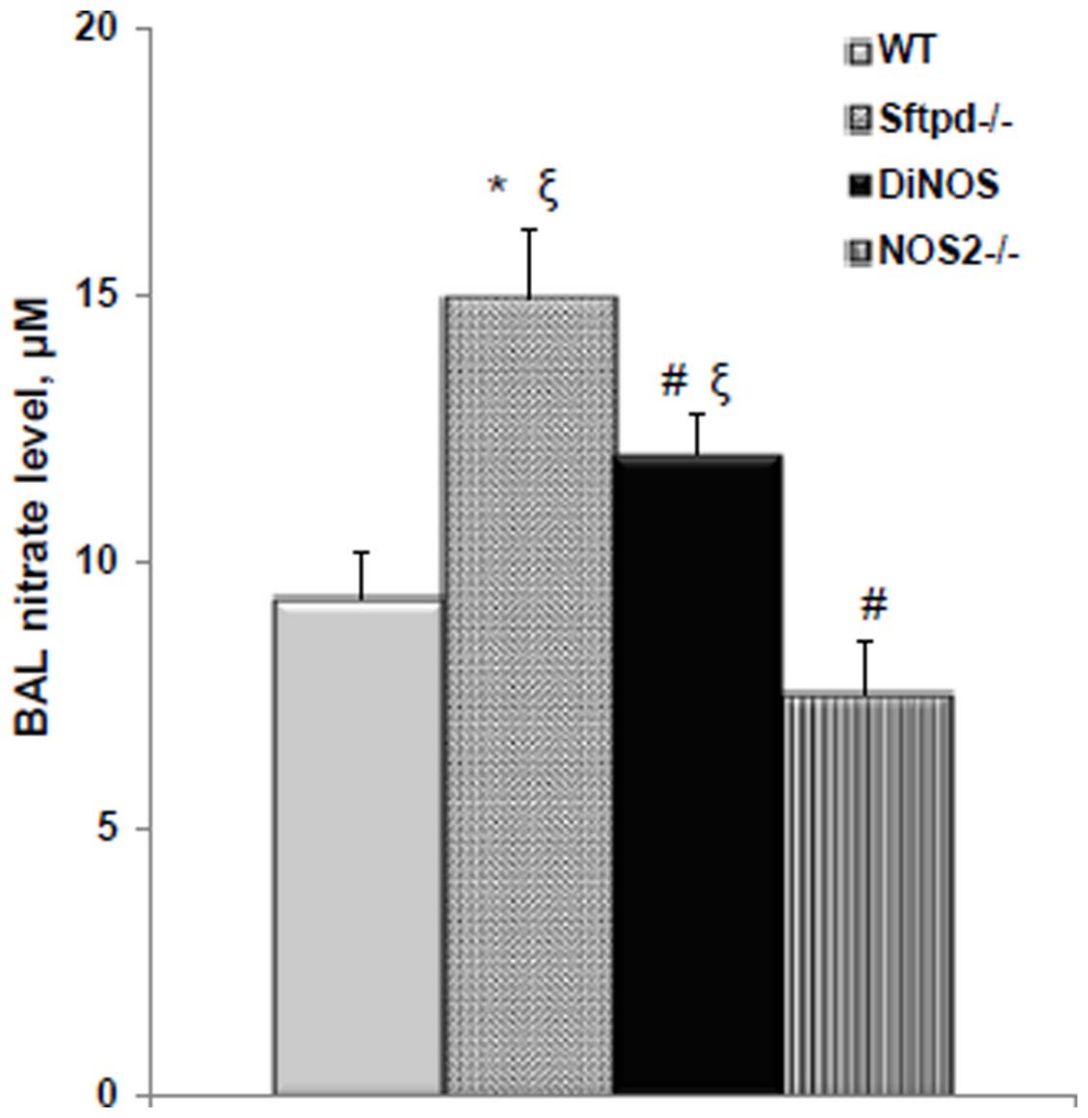
Ablation of NOS2 reduces NO metabolite production in DiNOS mice. BAL nitrates were measured by NOA as a marker of NOS activity. Values are mean ± S.E. (n = 6 to 15). Statistically significant differences between groups (p<0.05) are indicated as: * *vs* WT; ξ *vs* NOS2^−/−^; # *vs* Sftpd^−/−^ mice.

Activation of inflammation within the lung generally leads to increased NO production, principally via iNOS. Measurement of nitrate, a stable oxidative product of NO, within the BAL can be used as a marker of NO production within the lung. Sftpd^−/−^ mice have increased NO production relative to both WT and NOS2^−/−^ mice (**Figure 1**). In DiNOS mice the increase in BAL nitrates is reduced but not to control levels. These data are consistent with reduced NO production as a function of the loss of iNOS. However, they do highlight that BAL nitrates may be produced from alternate sources as the DiNOS level is still higher than NOS2-/- alone.

### Parenchymal inflammation in Sftpd^−/−^ mice is reduced in DiNOS mice

**Figure 2:**
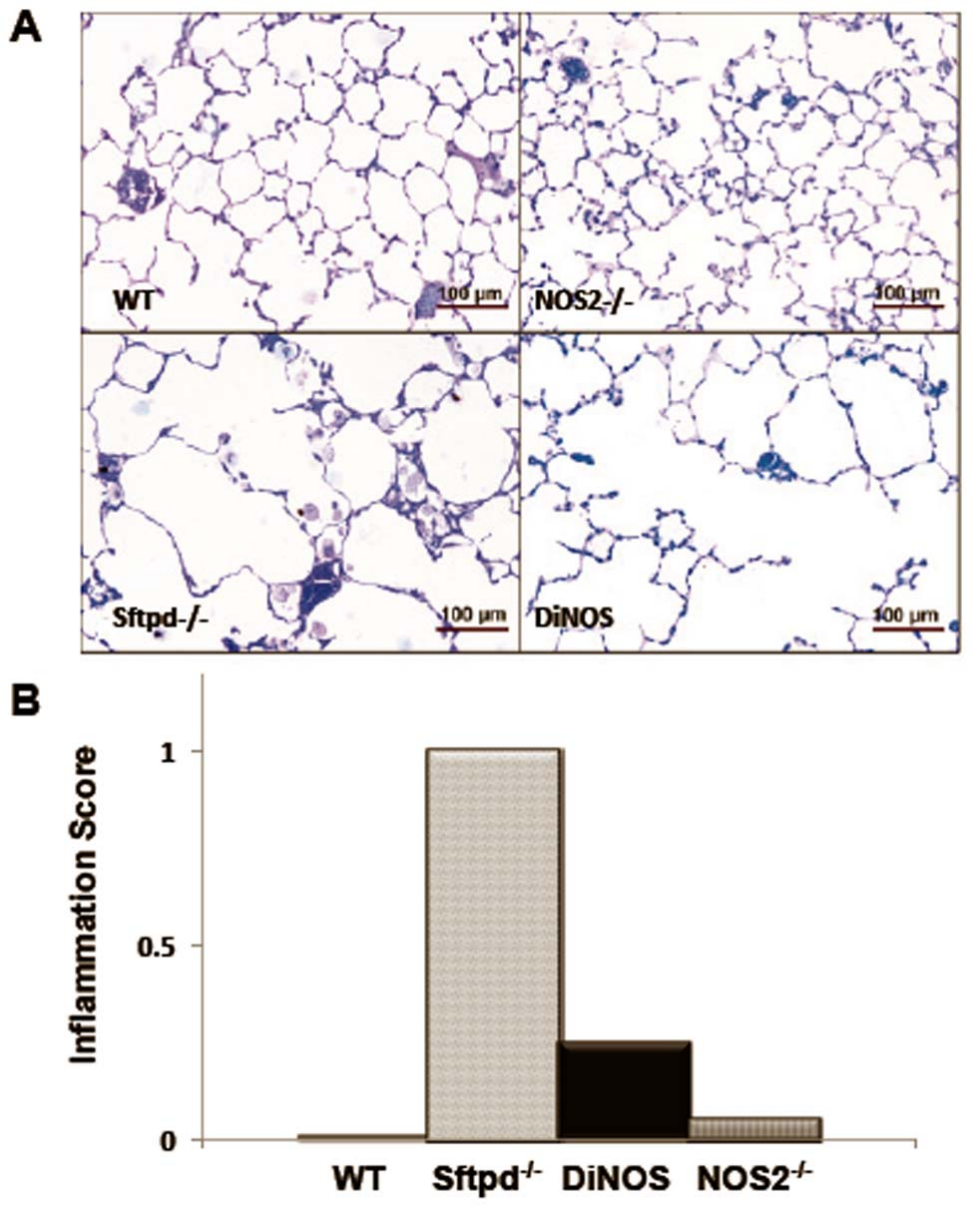
Lung histology reveals parenchymal inflammation in Sftpd^−/−^ mice and reduced enlargement of distal airspaces in DiNOS. (A) Representative micrographs of lung sections from WT (upper left), NOS2^−/−^ (upper right), Sftpd^−/−^ (lower left) and DiNOS (lower right) mice (toluidine blue staining). Normal lung architecture can be seen in WT and NOS2^−/−^ mice. In Sftpd^−/−^ the distal airspaces are enlarged and filled with foamy appearing alveolar macrophages. Enlargement of distal airspaces appears to be less pronounced in DiNOS compared to Sftpd^−/−^ mice. (B) Histological scoring of lung inflammation. Median inflammation scores were determined by blinded evaluation of stained sections from each genotype group as described in Online Supplement Methods. Data shown are Median, n = 7 animals per genotype.

Peribronchial and perivascular inflammatory infiltration, hypertrophy and hyperplasia of AE2 cells and an accumulation of macrophages in the BAL occurs in Sfptd-/- mice. At the light microscopic level WT and NOS2^−/−^ mice display slim alveolar walls and appropriately inflated distal airspaces with no signs of airspace enlargement or inflammatory cell accumulation (**Figure 2A**). In contrast, Sftpd^−/−^ mice are characterized by heterogeneous and focal enlargements of distal airspaces; while an intermediate airspace phenotype occurs in DiNOS mice. Accumulations of alveolar macrophages and intra-alveolar surfactant were occasionally found in these groups. These alterations were predominantly encountered in subpleural and peribronchial regions. In keeping with the magnitude of airspace enlargement (DiNOS mice less pronounced than the Sftpd^−/−^) the accumulation of macrophages and surfactant followed a similar pattern. These observations are confirmed by qualitative inflammatory scoring (**Figure 2B**).

### Ultrastructural evaluation of lung architecture

**Figure 3:**
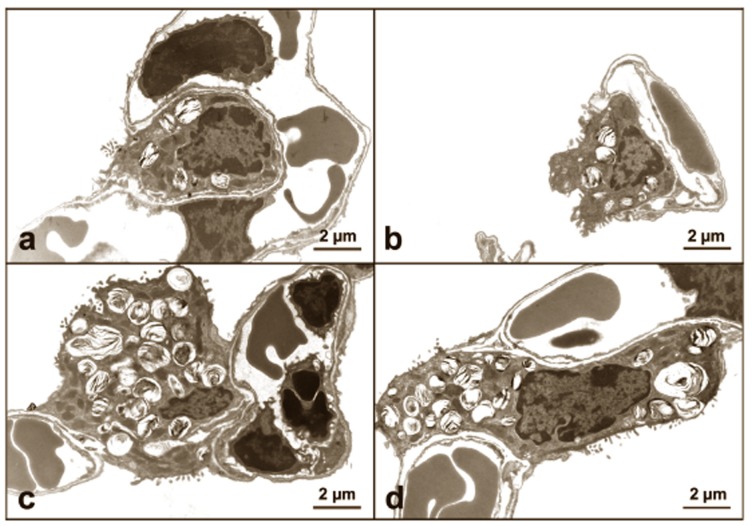
Lungs of DiNOS and Sftpd^−/−^ mice exhibit AE2 hyperplasia and hypertrophy with increased numbers of lamellar bodies. Representative electron micrographs of AE2 cells of WT (**a**), NOS2^−/−^ (**b**), Sftpd^−/−^ (**c**) and DiNOS (**d**) mice. The profiles of AE2 cells in Sftpd^−/−^ and DiNOS are bigger and seem to contain more lamellar bodies compared to WT and iNOS^−/−^ mice.

**Figure 4:**
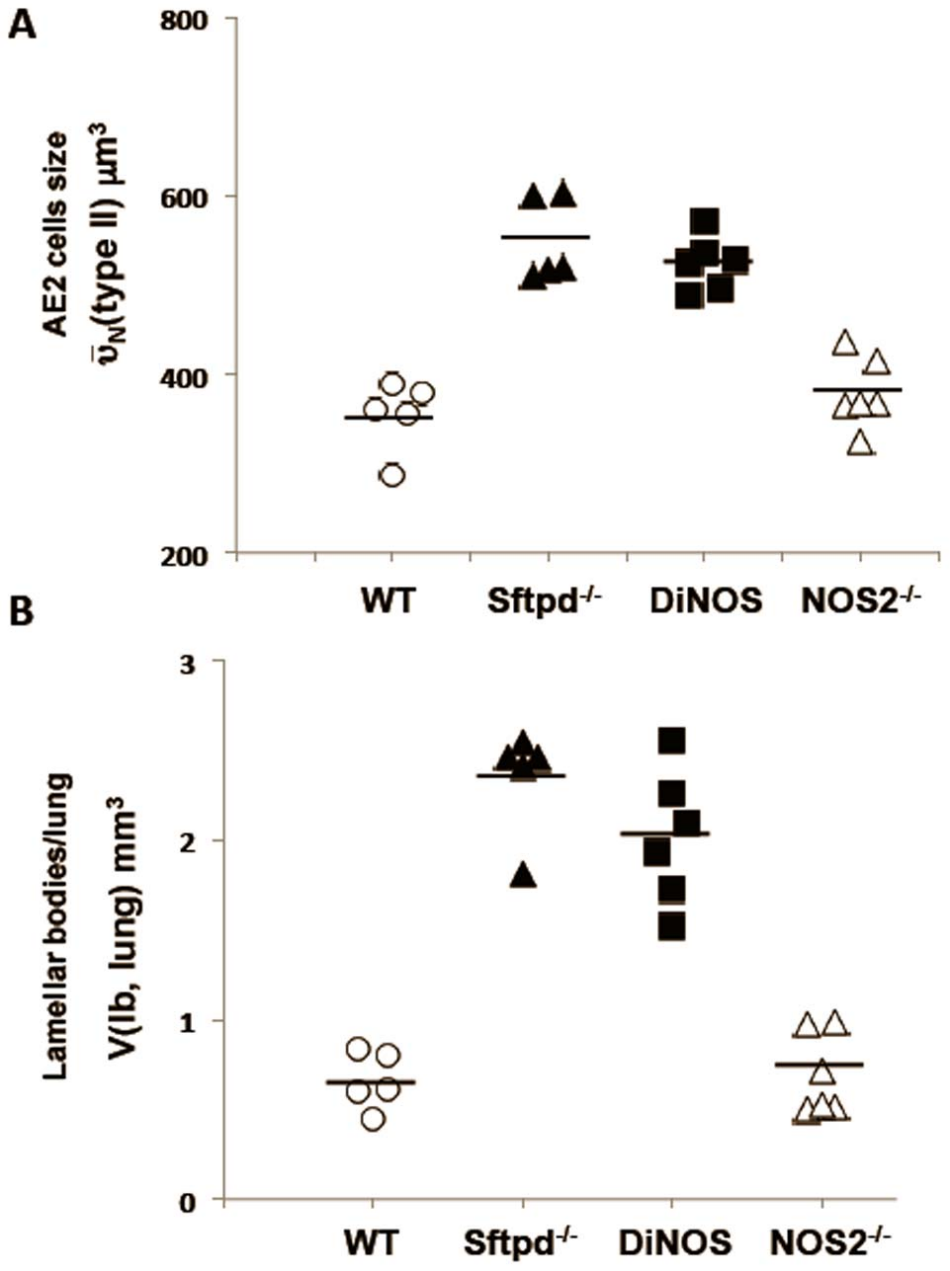
NOS2 ablation does not correct AE2 cell size and surfactant per lung in the absence of SP-D. Hypertrophy of AE2 cells (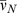 (typeII)) is present in Sftpd^−/−^ and DiNOS to a similar extent (**A**). The increased volume of lamellar bodies per lung V(lb,lung) (**B**) is unaffected by the additional ablation of the NOS2-gene in Sftpd deficient mice. Data shown are mean and individual data, n = 5–6 animals per genotype. Statistically significant differences between groups are shown in Table 3.

**Figure 5:**
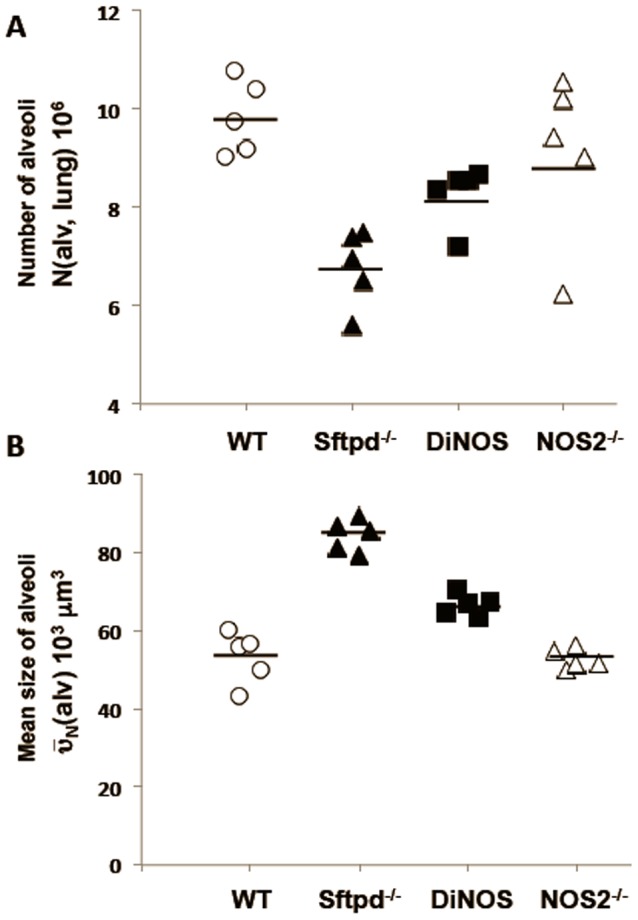
NOS2 ablation improves emphyatous phenotype resulting from the absence of SP-D. The number of alveoli per lung N(alv, lung) (**A**) is increased whereas the number-weighted mean volume of alveoli 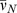 (alv) (**B**) is decreased in the DiNOS mice compared to Sftpd^−/−^ mice, indicating an attenuation of the pulmonary emphysema due to the additional ablation of the iNOS-gene in Sftpd deficient mice. Data shown are mean and individual data, n = 5–6 animals per genotype. Statistically significant differences between groups are shown in Table 2.

More and larger AE2 cells were observed on histologic examination of Sftpd^−/−^ and DiNOS mice when compared with both WT and NOS2^−/−^, indicating hyperplasia and/or hypertrophy (**Figure 3**). At the electron microscopic level, AE2 cells of Sftpd^−/−^ and DiNOS mice contained more surfactant material as indicated by lamellar body number (**Figure 3**). In both Sftpd^−/−^ and DiNOS mice giant lamellar bodies were occasionally observed. To further characterize the morphological changes associated with loss of SP-D and iNOS we conducted a series of stereological analyses (**Figures 4** and **5** and **Table 1**). The complete stereological results are presented in the online supplement. Regarding the total lung volume V(lung) no significant differences were observed between the genotypes.

**Table 1:** Stereological data of lung architecture.

Parameter	WT	Sftpd^−/−^	DiNOS	NOS2^−/−^
**V(lung) [cm^3^]**	1.17**±**0.03	1.20**±**0.05	1.12**±**0.03	1.05**±**0.06
**N(alv, lung) [10^6^]**	9.83**±**0.34	6.78**±**0.34* ξ	8.14**±**0.25	8.92**±**0.64
**N_V_(alv/par) [10^3^/mm^3^]**	9.66**±**0.28	6.58**±**0.16* ξ	8.21**±**0.21* ξ #	9.80**±**0.21
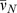 **(alv) [10^3^ µm^3^]**	53.2**±**3.0	84.5**±**1.9* ξ	66.8**±**1.0* ξ #	53.4**±**1.2
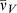 **(alv) [10^3^ µm^3^]**	152.4**±**13.7	342.0**±**23.3* ξ	217.1**±**25.0ξ #	107.6**±**9.28
**S(alvepi, lung) [cm^2^]**	734.7**±**39.4	568.5**±**27.3*	623.0**±**24.5	654.7**±**29.1
**S_V_(alvepi/par) [cm^2^/cm^3^]**	719.5**±**21.0	551.7**±**13.4* ξ	629.1**±**22.3* ξ #	726.4**±**19.5

Values are given as mean ± S.E. of n = 5–6 mice per genotype. Abbreviations: V = volume, S = surface area, S_V_ = surface area density, N = number, N_V_ = numerical density, 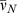 = number-weighted mean volume, 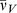 = volume-weighted mean volume, par = parenchyma, alvepi = alveolar epithelium, alv = alveoli. Statistically significant differences between groups (p<0.05) are indicated as:

* *vs.* WT,

ξ *vs.* NOS2^−/−^,

# *vs.* Sftpd^−/−^.

Our data show a persisting hyperplasia and hypertrophy of AE2 cells in the lungs of DiNOS, which did not differ from Sftpd^−/−^ mice. The number of AE2 per lung (N(typeII, lung), **Table 2**, as well the number-weighted mean volume (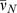 (typeII)) of AE2 cells was increased in these two groups compared to WT and NOS2^−/−^ mice (**Figure 4A**). In addition, the disturbances of the intracellular surfactant pool remained unaffected by the additional ablation of the NOS2 gene in Sftpd deficient mice (**Figure 4B**). The volume fraction of lamellar bodies within AE2 cells (V*_V_*(lb/typeII)) was increased in DiNOS and Sftpd^−/−^ mice to an equal extent, consecutively leading to an increase in the absolute volumes of lamellar bodies per AE2 cell (V(lb,typeII)) and within the whole lung (V(lb,lung). The total volume of lamellar bodies per AE2 cell was approximately doubled in both DiNOS and Sftpd^−/−^ mice compared to WT (**Table 2**).

**Table 2:** Stereological data: AE2 cells and intracellular surfactant.

Parameter	WT	Sftpd^−/−^	DiNOS	NOS2^−/−^
N(typeII,lung) [10^6^]	9.65±0.38	13.9±0.66* ξ	12.9±0.82* ξ	8.99±0.67
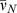 (typeII) [µm^3^]	354.6±18.0	550.8±21.1* ξ	525.0±12.2* ξ	379.1±16.1
V_V_(lb/typeII) [%]	19.2±1.50	30.6±1.19* ξ	29.7±0.57* ξ	20.1±1.05
V(lb,typeII) [µm^3^]	68.0±5.95	168.4±9.70* ξ	155.5±3.46* ξ	77.3±7.0
V(lb,lung) [mm^3^]	0.66±0.07	2.34±0.13* ξ	2.02±0.15* ξ	0.70±0.09
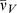 (lb) [µm^3^]	0.69±0.07	1.00±0.14	0.75±0.05	0.63±0.04

Values are given as mean ± S.E. of n = 5–6 mice per genotype. Abbreviations: V = volume, V_V_ = volume fraction, 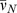 = number-weighted mean volume, 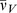 = volume-weighted mean volume, lb = lamellar body, type II = AE2. Statistically significant differences between groups (p<0.05) are indicated as:

* *vs.* WT,

ξ *vs.* NOS2^−/−^,

# *vs.* Sftpd^−/−^.

Regarding parameters characterizing emphysematous alteration of lung architecture, differences between Sftpd^−/−^ and DiNOS were observed. The surface density (S_V_(alvepi/par), total surface area (S(alvepi, lung)) of the alveolar epithelium, and the total number of alveoli per lung (N(alv,lung)) were markedly reduced in Sftpd^−/−^ compared to WT (**Table 1**). The DiNOS group compared to Sftpd^−/−^ on the one hand and WT on the other hand, showed increased but not normalized values regarding S(alvepi,lung) and N(alv,lung) indicating an attenuation of the emphysematous phenotype (**Figure 5A**). Taking the number-weighted mean volume of alveoli (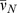
(alv)) into consideration, smaller alveoli were found in DiNOS mice compared to Sftpd^−/−^ mice, although the alveoli were still enlarged compared to WT (**Figure 5B**). These findings were confirmed by the volume-weighted mean volume of alveoli (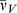
(alv)), a parameter feeling the heterogeneity of emphysematous alterations within the lung [29]. Thus, the additional ablation of the NOS2 gene improved emphysematous alterations in the Sftpd^−/−^ mouse whereas hyperplasia and hypertrophy of AE2 cells as well as the disturbances of the intracellular surfactant pool remained unaffected.

### NOS2 ablation normalizes lung resistance and elastance

**Figure 6:**
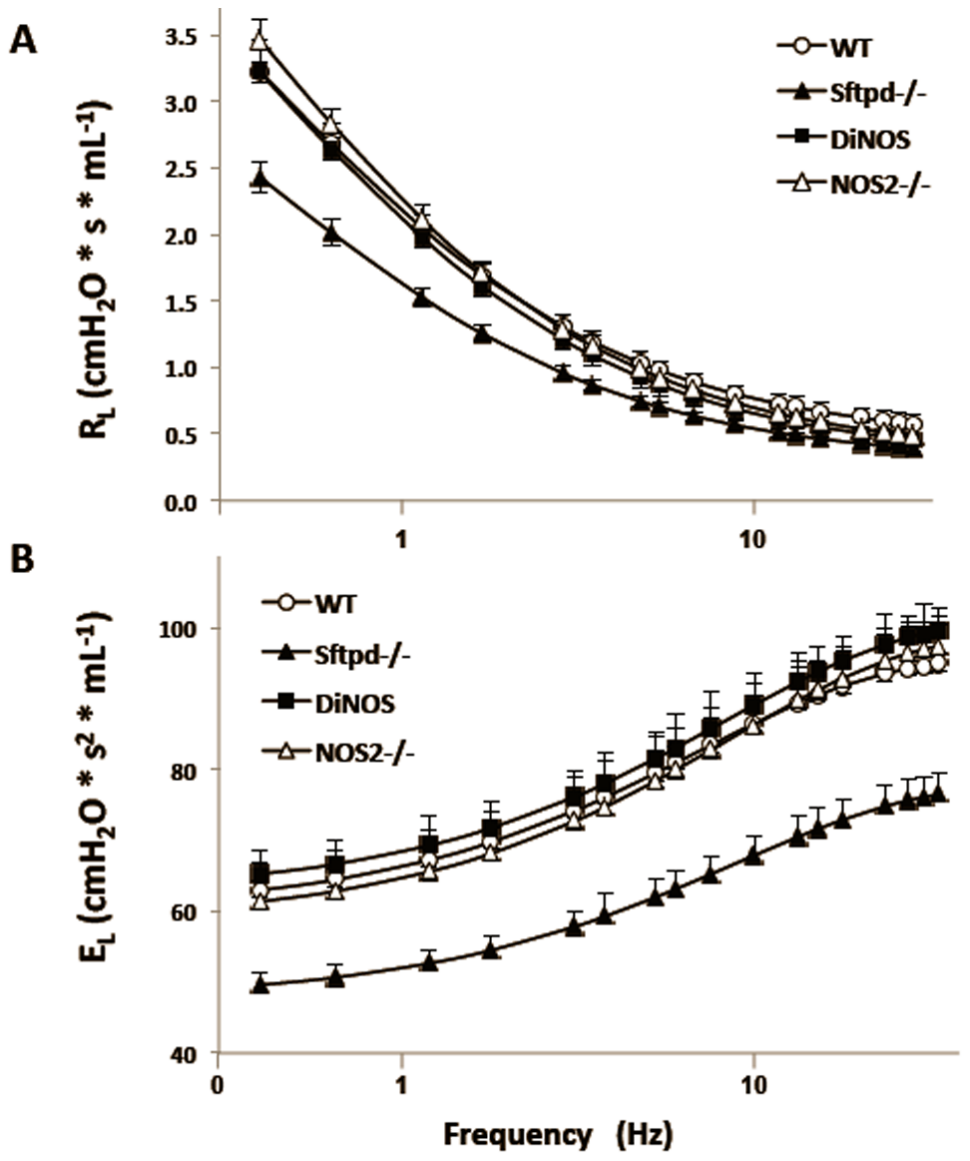
NOS2 ablation corrects lung resistance and elastance changes seen in Sftpd^−/−^ mice. Lung resistance (**A**) and elastance (**B∫**) spectra as a function of frequency, at a positive end-expiratory pressure (PEEP) of 3 cm H_2_O, are shown. Each point represents the mean of 3–6 measurements ± S.E., lines are the empirical fit [28] using the model outlined in the Online Supplement. Sftpd^−/−^ spectra are significantly different from WT as determined by χ^2^ test (p<0.05).

In order to examine the effects of loss of SP-D and iNOS on lung function, 12-week-old mice were analyzed by the Forced Oscillation Technique. From this measurement resistance (*R_L_*) and elastance (*E_L_*) spectra were analyzed by an empirical model that allows for physiological parameters to be estimated (28) (**Figure 6**). This model is preferred over the constant phase model, which assumes that the lung is ventilated homogenously [30]; an approximation that breaks down in pathological situations [31]. This is of particular concern with the Sftpd^−/−^ mouse, where there is heterogeneity in the lung restructuring and altered biophysics in the alveoli. Mean spectra were compared to WT by χ^2^ test to determine if there is a significant alteration in mechanical properties. Sftpd^−/−^ mice have a significantly lower resistance spectrum, however WT, NOS2^−/−^ and DiNOS do not significantly differ. Examination of the model fit parameters reveals that the low frequency portion of the spectrum determines this change (**Table 3**). There is no significant difference between any of the genotypes in the parameter b, which is determined by high frequency behavior; while a/c, which is determined by low frequency components is significantly lower in the Sftpd^−/−^ group.

**Table 3:** Lung function data: Parameter values from model fits.

	a/c	E_0_	ΔE
**WT**	3.57±0.39	60.07±3.66	35.59±1.97
**Sftpd** ^−/−^	2.37±0.08*	47.26±1.66*	31.51±1.64*
**DiNOS**	3.15±0.17	68.97±1.94*	38.35±1.36
**NOS2** ^−/−^	3.16±0.12	58.39±4.14	39.69±1.79

Values given are mean ± S.E. (n = 5–6) for derived parameters from individual R_L_ and E_L_ spectra. Significance comparisons are indicated by (*p<0.001 *vs.* WT).

The mean elastance spectrum of Sftpd^−/−^ mice is significantly lower than WT, while there is no significant difference between WT, NOS2^−/−^, and DiNOS (**Figure 6**). The only parameter that is significantly altered in the Sftpd^−/−^ genotype is E_0_, which represents the low frequency component of the lung. Neither ΔE, which represents the change in elastance with increasing frequency, or β, which is determined by the rate of change in stiffness with respect to frequency, displayed any significant change. These data can be best explained by a reduction in the parenchymal stiffness and resistance in Sftpd^−/−^ mice; consistent with the stereological observations of increased alveolar size and reduced alveolar number. Furthermore, these changes are not observed in DiNOS mice, consistent with the correction observed in their stereology.

### NOS2 ablation partially normalizes BAL cell counts

**Figure 7:**
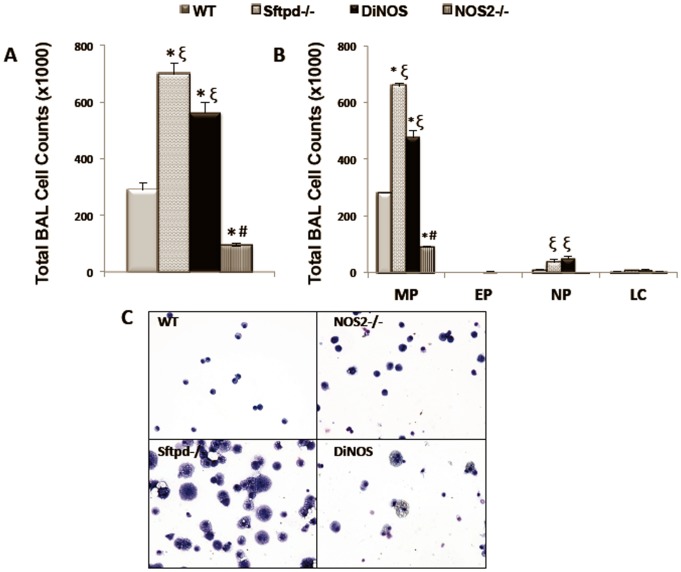
DiNOS mice exhibit partial normalization of BAL cell counts. Lungs were lavaged with 0.5-ml aliquots of sterile saline to a total of 5 ml. Recovered BALF samples were centrifuged (300 g for 10 min) and the cell pellet was gently resuspended in 1 ml of PBS (with Ca^2+^ and Mg^2+^). (**A**) Total cell count was determinated using a Z1 Counter particle counter (Beckman Coulter). (**B**) Aliquots of cells were spun on a Thermo Shandon Cytospin-3 at 750 rpm for 3 min and stained with standard Diff-Quik for manual determination of cell differentials. Cells were identified as macrophages, eosinophils, neutrophils, and lymphocytes by standard morphology. Data shown are mean ±S.E., n = 7 animals per genotype. Statistically significant differences between groups (p<0.05) are indicated as: * *vs* WT; ξ*vs* NOS2^−/−^; # *vs* Sftpd^−/−^ mice. (**C**) Representative Diff-Quik staining of cytospins from WT and Sftpd^−/−^, DiNOS and NOS2^−/−^ mice.

The ultrastructure and physiological data suggested a significant impact of iNOS signaling on the lung phenotype of Sftpd^−/−^ mice. Examination of cells from lung lavage reveals that, as previously reported, there is an increase in cell number in Sftpd^−/−^ mice (**Figure 7A**). Cell numbers are significantly lower in DiNOS mice. However, NOS2^−/−^ cell numbers are similarly reduced relative to WT, implying this may be a general effect of NOS2 ablation rather than a specific effect related to the loss of SP-D. Examination of cell differentials via Giemsa stain reveals that the reduced cell number in NOS2^−/−^ and DiNOS mice is reflected in reduced macrophage numbers; while there is a slight increase in neutrophil number within DiNOS mice relative to Sftpd^−/−^ (**Figure 7B**). Despite the reduction in the number of recruited macrophages in DiNOS mice, the large and foamy macrophages seen in Sftpd^−/−^ mice are still observed (**Figure 7C**).

### Surfactant Homeostasis Is Not Corrected by Additional NOS2 Ablation in DiNOS Mice

One possible explanation for the consistent macrophage morphology in DiNOS and Sftpd^−/−^ mice, is that ablation of the NOS2 gene did not correct the lipoproteinosis observed with the loss of the Sftpd gene [6], [7]. Analysis of the BAL fluid for phospholipid content reveals this to be in part correct (**Table 4**). The total phospholipid content of both DiNOS and Sftpd^−/−^ mice is greater than that of WT and NOS2^−/−^. However, the increase seen in phospholipid content within Sftpd^−/−^ mice occurs in both the small and large aggregate fractions. Within DiNOS mice the increase within the large aggregate fraction is not changed. In contrast within the small aggregate fraction where phospholipid content is increased approximately 8 fold to 604 µg within Sftpd^−/−^ mice, it is increased only 6 fold to 465 µg in DiNOS mice. The large aggregate fraction of the BAL contains the bulk of the surface-active material. These data are consistent with ablation of the NOS2 reducing the inflammatory effects of SP-D knockout but only minimally impacting surfactant pool sizes.

**Table 4:** Surfactant homeostasis is not corrected by additional NOS2 genetic ablation.

	Surfactant Fraction		
	Small (µg)	Large (µg)	Total (µg)
**WT** (n = 7)	76±6	148±10	224±15
**Sftpd^−/−^** (n = 15)	604±39 * ξ	249±15 * ξ	854±49 * ξ
**DiNOS** (n = 7)	465±34 * ξ #	233±18 * ξ	698±42 * ξ
**NOS2^−/−^** (n = 8)	116±5 #	112±6 #	228±9 #

Phospholipid content of BAL as well as small and large aggregate fractions was determined by the method of Bartlett. Data shown are mean ± S.E.. Statistically significant differences between groups (p<0.05) are indicated as:

* *vs.* WT,

ξ *vs.* NOS2^−/−^,

# *vs.* Sftpd^−/−^ mice.

### NOS2 Ablation Alters the Inflammatory Phenotype of Alveolar Macrophages

**Figure 8:**
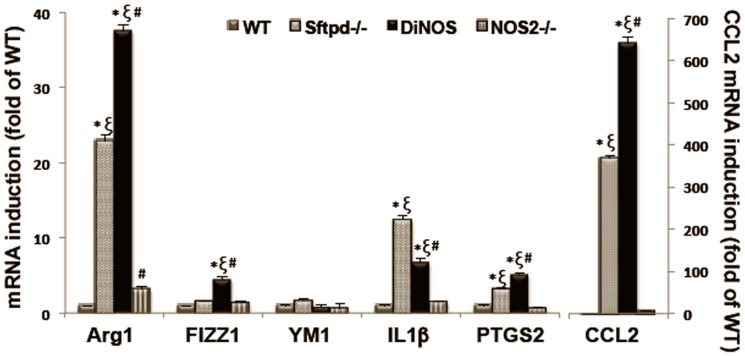
NOS2 ablation alters the inflammatory phenotype of BAL in DiNOS mice. RNA was extracted from BAL cells isolated from WT, Sftpd^−/−^, NOS2^−/−^ and DiNOS mice. Gene markers were quantified by RT-qPCR as described. Ct values obtained were normalized to β-actin signals and further analyzed using the relative quantization (ΔΔCt) method. Data are expressed as fold change (means ± S.E.M, n = 5–8 in each group). Statistically significant differences between groups (p<0.05) are indicated as: * *vs* WT, ξ *vs* NOS2^−/−^, # *vs* Sftpd^−/−^.

Having established that ablation of the NOS2 gene did not appear to affect AE2 cell morphology (**Figure 3** **,** **Table 1**) but did reduce inflammatory cell recruitment to the lung (**Figure 7**), we examined the cell pellet from the BAL for expression of inflammatory markers (**Figure 8**). The induction of NOS2 itself is often used as a marker of inflammatory activation, as is the case in Sftpd^−/−^ mice. In the absence of the NOS2 gene, we examined IL-1β expression as a general marker of activation [32]. IL-1β expression was significantly increased in Sftpd^−/−^ mice, however this increase was halved in DiNOS animals. In contrast, PTGS2, which was increased in Sftpd^−/−^ mice, was not altered by the loss of NOS2. ARG1, FIZZ1, and CCL2 all of which are markers of alternative macrophage activation [33], are increased with loss of SP-D and this increase is enhanced in DiNOS. Ym1, which along with ARG1 has been identified as a marker of alternative myeloid activation in murine cells [34], was not significantly altered in terms of fold expression, however, it is notable that baseline Ym1 expression is high in the lung (ΔCt). These data indicate that there appears to be a shift in inflammatory cell signaling within DiNOS mice.

## Discussion

The crucial role of increased iNOS activity in the emphysematous remodeling of mouse lungs has been revealed recently and this pathway may be relevant to human COPD [35], as cigarette smoke reduces SP-D levels in human BAL [36], [37]. The lack of SP-D, moreover, results in an iNOS mediated chronic inflammation in mice [11]. In this study we have examined whether ablation of the NOS2 gene rectifies the surfactant and structural anomalies observed in Sftpd^−/−^ mice. Previously, we observed that transient inhibition of iNOS reduced inflammation in Sftpd^−/−^ mice without altering the lipoproteinosis [38]. Here, we see that in mice lacking both genes, DiNOS, there is a reduction in inflammation that is accompanied by a correction of the alveolar structural abnormalities. However, AE2 cell hyperplasia is maintained, as is the excessive production of surfactant. Notably the alterations in lung function within Sftpd^−/−^ can be observed using a Forced Oscillation Technique [39]. Here, we have extended these observations to an Empirical model, which demonstrates a considerable reduction in low frequency resistance and lung “stiffness” at the static limit in Sftpd^−/−^ mice. Despite the significant surfactant abnormalities within DiNOS mice, lung function appears to be fully restored. These observations highlight the importance of iNOS in mediating the inflammation that occurs within Sftpd^−/−^ mice and that these processes form the basis of the structural and functional abnormalities that occur.

The Sftpd^−/−^ mouse has been shown to have an emphysematous phenotype, largely on the basis of qualitative histological examination [38], [40], [41]. In this study we have conducted a detailed stereological analysis to characterize the structural abnormalities that occur within Sftpd^−/−^ mice. These studies revealed that while there is no change in overall lung volume there is an increase in mean alveolar size and a decrease in the total number of alveoli per lung; and thus an overall loss of alveolar surface area. These studies provide a quantitative measure of the structural alterations that occur in Sftpd^−/−^ mice and allow for measurement of the pathology.

Ablation of the SP-D gene results in a significant lipoproteinosis [42], and in accordance with this disruption, we have observed an increase in AE2 cell volume and the quantity of surfactant stored (**Figure 4**), indicating both a hyperplasia and a hypertrophy (**Figure 3**). Further, macrophages in the lung lining are large and foamy, presumably due to excessive phagocytosis of lipid. These data implicate excessive production of the surface-active component of the lung lining by AE2 cells. However, the greatest increase in phospholipid content occurs within the small aggregate fraction of the BAL rather than the large aggregate. This fraction typically consists of the non-surface active components, such as the pulmonary collectins, and is associated with innate immune function. Therefore, it seems that loss of SP-D results in disruption of both the inflammatory and the surfactant functions of the pulmonary epithelium.

iNOS inhibition reduces the inflammation but not the lipoproteinosis observed in Sftpd^−/−^ mice [38]. The creation of the DiNOS mouse, along with sophisticated stereology, has allowed us to quantify the effects of the loss of iNOS function upon the Sftpd^−/−^ mediated structural abnormalities. Importantly, loss of the NOS2 gene has similar effects on pulmonary inflammatory markers to those observed with iNOS inhibition. The inflammatory score is reduced, as is total cell count, and specifically the number of macrophages, within the lung lining. Also the production of NO metabolites falls, although not to control levels indicating the importance of other sources, such as nNOS, on BAL nitrate levels. Stereological analysis reveals normalization of the mean alveolar size and the number of alveoli per lung number of measures of the structural abnormality seen in Sftpd^−/−^ mice (**Figure 5** and **Table 1**). These changes are apparent at the qualitative level when examining the histology of the lung (**Figure 2**).

In DiNOS mice there is little change either to the AE2 cell or macrophage morphology relative to Sftpd^−/−^ (**Figure 3** **and** **Figure 7C**). This is further reflected in a failure of iNOS ablation to reduce either the increased number of AE2 cells or the increased volume of surfactant within the lung (**Table 1**). These observations are consistent with previous studies where inhibition of iNOS function for two weeks reduced pulmonary inflammation without altering the surfactant profile [38]. However, the phospholipid content of the small aggregate fraction of the lung lining fluid is reduced in DiNOS (**Table 4**), while the large aggregate fraction is unaffected. The large aggregate fraction consists of the surface-active components phophatidyl choline, phophatidyl serine and the surfactant proteins B & C, while the small aggregate fraction compromises unincorporated lipid and the immunoregulatory molecules such as SP-A & D [43]. That the small aggregate function is normalized to some extent by loss of NOS2, while the large aggregate fraction is unaffected, emphasizes the role of iNOS as a mediator of pulmonary inflammation.

The Forced Oscillation Technique is an accepted method to examine lung function at the organ level. Such data it is usually fit to the constant phase model [44], which allows for comparison of proposed physiological parameters. We have developed an empirical model that while losing the advantage of a proposed physiological relevance to its parameters gains in its accuracy to non-homogenous lung function [28]. In agreement with previous work [39], observation of both the *E_L_* and the *R_L_* spectra show that there is a major alteration in lung function within Sftpd^−/−^ mice (**Figure 6**). Parameter analyses demonstrate a reduction in low frequency resistance and in elastance at the static limit within Sftpd^−/−^ mice (**Figure 6**). These data are best explained by an alteration in the inherent mechanical properties of the lung. The high frequency component of both the resistance and elastance spectra are not significantly altered when compared to WT. Examination of the stereology and the lipoproteinosis offer possible explanations for this mechanical behavior. The increased mean alveolar size and reduced alveolar number seen in Sftpd^−/−^ mice could result in reduced low frequency mechanical parameters. The determination of alveolar number is based on the determination of the connectivity of the axial elastic fiber network involved in forming alveolar entrance rings [8], [45], [46]. Thus, a reduction of alveolar number is associated with a decrease in the connectivity of this axial elastic fiber network, which might result in softening of fine lung parenchyma in Sftpd^−/−^ mice and subsequently decrease lung elastance at its static limits. Whilst altered surfactant function could also result in reduced lung “stiffness” and a reduction in low frequency resistance due to altered parenchymal tethering. In DiNOS mice there is a resolution of the structural abnormalities without an improvement in the surfactant profile; and in these mice the functional abnormalities appear to be resolved. Therefore, one can conclude that the changes observed at the organ level observed within Stfpd^−/−^ mice are a result of the alveolar restructuring rather than a result of the alveolar proteinosis.

In summary, these data demonstrate that increased iNOS activity is critical to the remodeling of alveolar architecture and related mechanical properties of lung parenchyma in Sftpd^−/−^ mice whereas disturbances of surfactant homeostasis are independent of iNOS. Shifting of the function of alveolar macrophages towards an alternative activation with anti-inflammatory properties might explain these observations.
